# Plant community re-organization and increased productivity due to multi-year nutrient enrichment of a coastal grassland

**DOI:** 10.1371/journal.pone.0270798

**Published:** 2022-07-28

**Authors:** Joseph K. Brown, Ashley Moulton, Julie C. Zinnert

**Affiliations:** 1 Department of Biology, Virginia Commonwealth University, Richmond, Virginia, United States of America; 2 Department of Biological Sciences, Minnesota State University, Mankato, Minnesota, United States of America; Seoul National University, REPUBLIC OF KOREA

## Abstract

Nutrient enrichment alters plant community structure and function at a global scale. Coastal plant systems are expected to experience increased rates of nitrogen and phosphorus deposition by 2100, caused mostly by anthropogenic activity. Despite high density of studies investigating connections between plant community structure and ecosystem function in response to nutrient addition, inconsistencies in system response based on the ecosystem in question calls for more detailed analyses of nutrient impacts on community organization and resulting productivity response. Here, we focus on nutrient addition impacts on community structure and organization as well as productivity of different lifeforms in a coastal grassland. We established long-term nutrient enrichment plots in 2015 consisting of control (C), nitrogen (N), phosphorus (P), and nitrogen + phosphorus (NP) treatments. In 2017 we collected graminoid and forb productivity, root productivity, and community composition for each plot. We found no N x P interaction, but N enrichment was a significant main effect on productivity, highlighting N limitation in coastal systems. Importantly, nutrient enrichment treatments did not alter root productivity. However, all treatments caused significant differences in community composition. Using rank abundance curves, we determined that community composition differences were driven by increased dominance of nitrophilous graminoids, re-organization of subordinate species, and species absences in N and NP plots. Results of this study highlight how coastal grassland communities are impacted by nutrient enrichment. We show that community re-organization, increased dominance, and absence of critical species are all important mechanisms that reflect community-level impacts of nutrient enrichment in our coastal grassland site.

## Introduction

As global N and P enrichment becomes more common, researchers are investigating the effects nutrients have on plant communities around the world [1–3]. Increased nutrient enrichment can alter plant community composition, plant functional trait expressions, and ecosystem functionality [1, 3–8]. Nutrient enrichment influences species composition as well as community structure and organization (i.e., species diversity, richness, evenness, and dominance), often at large spatial scales [4, 7, 9–15]. However, scientific literature reflects a lack of consistency regarding community response to nutrient enrichment. For example, some studies show increased N and P enrichment results in decreased species diversity, evenness, and richness, with species disappearance identified as a common occurrence [12, 16–21], while others do not [22–24]. Nutrient enrichment commonly impacts primary productivity levels [1, 2, 5, 25], but some studies have shown no impact on forb or graminoid species [26]. Increased productivity after nutrient enrichment can result in higher competition for space and light, mechanisms recognized as drivers of altered community composition and structure [5, 11, 12, 18, 19, 27, 28]. Given tight connections between ecosystem productivity and community structure, it is important that existing variabilities are addressed to understand how nutrients impact plant communities in a variety of ecosystems [29].

The unpredictability of system response to nutrient enrichment highlights how differences in regional species pools and/or pre-existing environmental conditions may influence the final community structure and ecosystem functionality as a system responds to multiple nutrient enrichment treatments. Thus, it is critical to investigate how N and P (both individually and synergistically) influence plant community dynamics in coastal grassland ecosystems which have different abiotic stressors than other grasslands. Coastal grasslands occurring behind dune systems on barrier islands are uniquely impacted by environmental factors like sea-spray, varying access to freshwater, and sand burial that could influence plant community response to nutrient enrichment [30]. Furthermore, barrier islands are present globally in the same areas that are expected to increase in nutrient deposition [31]. Barrier islands occur along 30% of U.S. coastlines with more than half existing on the Atlantic coast where nutrient enrichment is expected to spike [31, 32]. Barrier island dynamics including disturbance response and resilience are impacted by feedbacks between dominant physical processes and extant plant communities [30, 33–35]. By investigating barrier island response to nutrient enrichments, it is possible to better understand how increased nutrient deposition impacts ecosystems experiencing multiple disturbances over short temporal scales. High nutrient enrichment via run-off and atmospheric deposition onto barrier islands can play a substantial role in driving plant community differences in composition of species and expressed functional traits. Impacts of nutrient enrichment may be heightened in coastal grasslands given the prominence of N limitation in sandy soils that serve as the foundation of barrier islands [8, 33, 36–38]. As a result, hypotheses have been established that attempt to explain how plant communities altered by nutrient enrichment leads to subsequent changes in barrier island ecology, specifically disturbance response and successional processes in grasslands developing behind dunes [13, 39].

In this study we focus on a mid-Atlantic coastal grassland, as this geographic region is likely to experience dramatic increases in nutrient loading in the next 80–100 years [31]. Previous nutrient enrichment models suggest coastal areas in the northern hemisphere have been dramatically impacted by N deposition in the last 30 years, with recent work highlighting the role chemically reduced forms of N has on global N flux [40–42]. Projections of future change show that, in the continental U.S., the Atlantic coast is expected to see the largest regional increase in N enrichment by 2100 [31]. Past nutrient enrichment studies in coastal systems have shown that N enrichment can cause long-term plant community change on sand dunes [37, 43, 44]. Day et al. [37] found that after N enrichment on a dune system, species re-ordering ensues with longstanding shifts in species dominance and species disappearance. Aboveground and belowground biomass were higher just one year after N fertilization treatment with effects lasting nearly 10 years [43]. Similar conclusions have been made in European dune systems where elevated nutrient deposition increases productivity and alters plant community structure, specifically reducing species richness [14].

Previous research in calcareous European sand dunes also shows that decalcified acidic sand dunes are more sensitive to N deposition due to the increased availability of P, highlighting the importance of both N and P for plant community response to expected nutrient enrichment [45]. Increases in P enrichment through run-off and deposition has accrued less focus but research has found that coastal systems are some of the most at-risk ecosystems for P enrichment [46]. For example, Mahowald et al. [47] suggests a net gain in atmospheric total P in terrestrial systems located near oceans, while most other terrestrial systems show a net loss of total P. Although it has been proposed P is not a limiting nutrient in terrestrial coastal systems [48–50], Kooijman et al. [45] showed sensitivity of dune vegetation to N deposition when P is abundantly available in the soil. Such findings justifies investigating N, P, and N + P (NP) treatments in coastal systems. Furthermore, N and P can influence plant communities synergistically, such that productivity is higher in NP treatments than either N or P treatments individually [1, 51]. Despite work on coastal sand dunes, knowledge gaps remain in understanding how non-dune coastal grassland systems will respond to expected nutrient enrichment.

We address these knowledge gaps by conducting a 3-year nutrient enrichment study to understand how prolonged N, P, and NP treatments affect coastal grassland communities. Specifically, we aim to understand how nutrient enrichment alters lifeform productivity and plant community composition after 3 years of nutrient treatments. In this study we focus on using plant community structure (evenness, richness, and diversity) and reorganization to explain altered community composition. We used visualization of rank abundance curves (RACs) to explore whether systematic plant community re-organization contributes to community differences [52]. We hypothesize that 1) both aboveground and belowground graminoid and forb productivity will increase with N and NP treatments, 2) that N and NP additions will cause differences in community structure metrics (species diversity, richness, and evenness), and 3) compared to control plots (serving as a reference of a natural community), N and NP, enrichment will cause differences in community composition. Based on previous findings, we do not expect P to have major impacts on biomass or community structure unless N enrichment is also present.

## Methods

### Study site, plot establishment, and nutrient application

**Fig 1:**
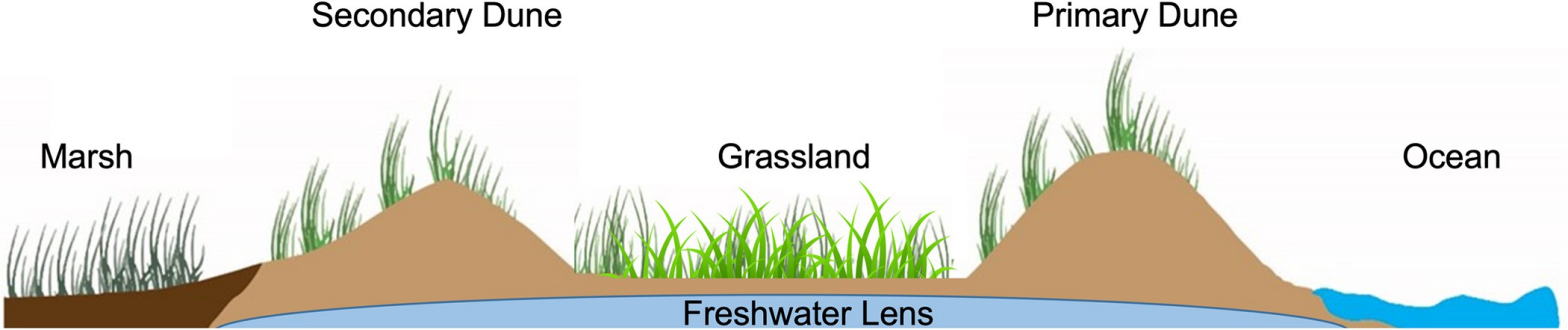
Cross-section schematic of barrier island.

Barrier islands can vary in morphological characteristics, but most have a foredune or series of dunes proceeding a marsh ecosystem on the backside with grasslands developing in lower lying areas between dunes (Fig 1). Access to freshwater for extant plant communities on barrier islands are reliant on a freshwater lens that is fed entirely by precipitation (Fig 1). Hog Island (37.417 N, 75.686 W) is a barrier island that is part of the Virginia Coast Reserve (VCR) Long-term Ecological Research site along the U.S. Atlantic coast. Nutrient fertilization plots were established on the southern end of the island in a grassland environment composed of perennial graminoid and annual/biennial forb species. The site consists of sandy, well-drained soils and has been described as stable due to presence of a linear dune ridge which provides protection from coastal disturbances [35].

Cross-section of a traditional barrier island morphology. Prominent landforms and habitats characteristic of a barrier island include a primary and secondary dune, with grasslands colonizing low lying areas between dune ridges. Plant communities in terrestrial barrier island habitats are dependent on a rain-fed freshwater lens. Terrestrial habitats eventually reach an ecotone where marsh species dominate, and semidiurnal tidal cycling is present.

Plots were established on the Hog Island grassland in 2015 following a modified Nutrient Network approach [53]. Experimental set-up followed a completely randomized block design with nutrient applications twice per growing season (May and June) from 2015–2017, we administered split fertilization application to enhance uptake efficiency by C3 and C4 species. Each nutrient treatment was administrated at 5 g m^-2^ yr^-1^, totaling 10 g m^-2^ yr^-1^ by the end of each season. The result of the experimental set-up was four treatments; nitrogen (N10 P0 or N), phosphorus (N0 P10 or P), nitrogen + phosphorus (N10 P10 or NP), and control (N0 P0 or C) with each treatment replicated five times (n = 5). Blocks were a total of 6 m^2^, treatment units within each block were 3 m^2^ with 1 m walkways, which were not recognized as usable plot space. Treatment units were further subdivided into four 1 m^2^ plots to allow for separate spaces for destructive productivity sampling and long-term species composition data collection. Phosphorus was applied as triple super phosphate, while N was applied as ammonium nitrate in particulate solid form.

### Lifeform productivity sampling

Productivity was defined as all aboveground vegetative biomass at the end of the growing season (September 2017). Productivity was sampled from one subplot within a haphazardly placed 0.1 x 1 m frame and extrapolated to represent plot level productivity (g m^-2^). Samples were sorted by lifeform (graminoid or forb), oven dried separately for 72 h at 60°C, and weighed (g). Belowground productivity was also collected in 2017. We used a single root core taken in a haphazardly selected spot in the same subplot where aboveground biomass was collected. For each root core, large roots were identified visually and collected from each soil core before the remainder of the cores were washed in a series of sieve stacks to collect smaller roots. All roots were rinsed with water before samples were oven dried for 72 h at 60°C and weighed (g).

### Species composition and community structure

In July of 2017 we recorded aerial species cover to the nearest 1% using an undisturbed subplot designated for species composition data collection. We calculated species evenness, richness, and diversity using the *codyn* package in R [54]. Species richness was computed as the number of different species in each replicate while species evenness was calculated as *Evar* for each replicate, this evenness index is recommended for general use compared to other evenness indices [55]. *Evar* produces an index between 0–1, where 0 represents minimum evenness within a replicate and 1 represents maximum evenness within a replicate [55]. Species diversity was calculated as Shannon’s diversity index for each replicate (H’).

Abundance differences were calculated for each species using control plots as a reference treatment. We pooled species abundance across all replicates to create a single species pool for our study site [54]. Developing a species pool allowed us to investigate how abundances of each species present at our sites differed between control plots and each treatment group. For example, a positive value indicated that a species had higher abundance in the treatment community than in the control community, while negative values indicated that a species had lower abundance in the treatment community than in the control community. Averaged species abundances were ranked in order of most abundant to least abundant to visualize the RACs for each treatment. RACs have been utilized in previous studies to inform differences in species compositions among treatment groups [7, 52].

### Statistical analysis

All analyses were conducted using R statistical program (R Core Team 2019). We used a two-way analysis of variance (ANOVA) to determine whether mean (± SE) graminoid and forb productivity were significantly impacted by N x P interaction (α = 0.05). Two-way ANOVAs were performed on log transformed productivity data to meet assumptions of normality (checked visually using QQ-plots) and homogeneity of variance (assessed using Levene’s test). Tukey HSD was used as a post-hoc test to determine significant pairwise differences among specific nutrient treatments (α = 0.05). A two-way ANOVA was used to test for a significant N x P interaction for community structure metrics (species diversity, richness, and evenness), after confirming assumptions of normality and homogeneity of variance. Tukey HSD was used as a pairwise post-hoc test for each community structure metric (α = 0.05).

Non-metric multidimensional scaling (NMDS) ordination was used to visualize community composition of nutrient enrichment treatments. Resulting NMDS visualization identifies replicate similarity based on distance in ordination space. Points (representing experimental replicates) closer together are more similar in community composition than points that are further apart. Prior to conducting the NMDS, species abundances were standardized to represent a relative abundance for each replicate. We conducted the NMDS in *vegan* R package using Bray-Curtis distance measure to calculate a distance matrix of relative species abundances for each treatment replicate [56]. The NMDS was performed using 3-dimensions to a minimized stress value (maximum iteration = 999). We used Monte Carlo randomization test to determine whether the final NMDS solution had a lower stress than would be expected by chance (α = 0.05). A principal components rotation was applied to the finalized NMDS to aid interpretation. This technique rotates the finalized plot such that NMDS axis 1 and 2 represent maximum variation of the data allowing for calculation of species correlations in ordination space.

We used the envfit function in *vegan* to calculate species correlations in ordination space [56]. Significance of fitted species correlations was assessed within the envfit function using permutation test (permutation = 999). Species that produced significant *p*-values are more correlated in ordination space than would be expected by chance and thus explain a large proportion of variation in the data. These species were highlighted in RACs to examine the impact of species rank order shuffling on community composition dissimilarity.

Centroids for each treatment group were calculated by aggregating site scores. Centroids can be interpreted as mean community composition for each treatment group. We used a permutational multivariate-ANOVA (PERMANOVA) to determine significant differences among community compositions of nutrient enrichment treatments (permutation = 999, α = 0.05) [57]. A pairwise post-hoc test was conducted to determine which pairwise treatment groups had different community compositions (FDR adjusted α = 0.05). We calculated multivariate homogeneity of group dispersion to determine within-group variation among treatment replicates using the betadisper function in *vegan* [56, 58]. Testing homogeneity of multivariate group dispersion has been identified as a distance-based method of identifying differences in beta-diversity among groups [59]. We used an ANOVA on mean distance between each point and their respective median centroid to determine significant differences of beta-dispersion among treatment groups (α = 0.05). A Tukey HSD test was used as a post-hoc test for pairwise differences in mean beta-dispersion (α = 0.05).

## Results

### Lifeform and belowground productivity

**Fig 2:**
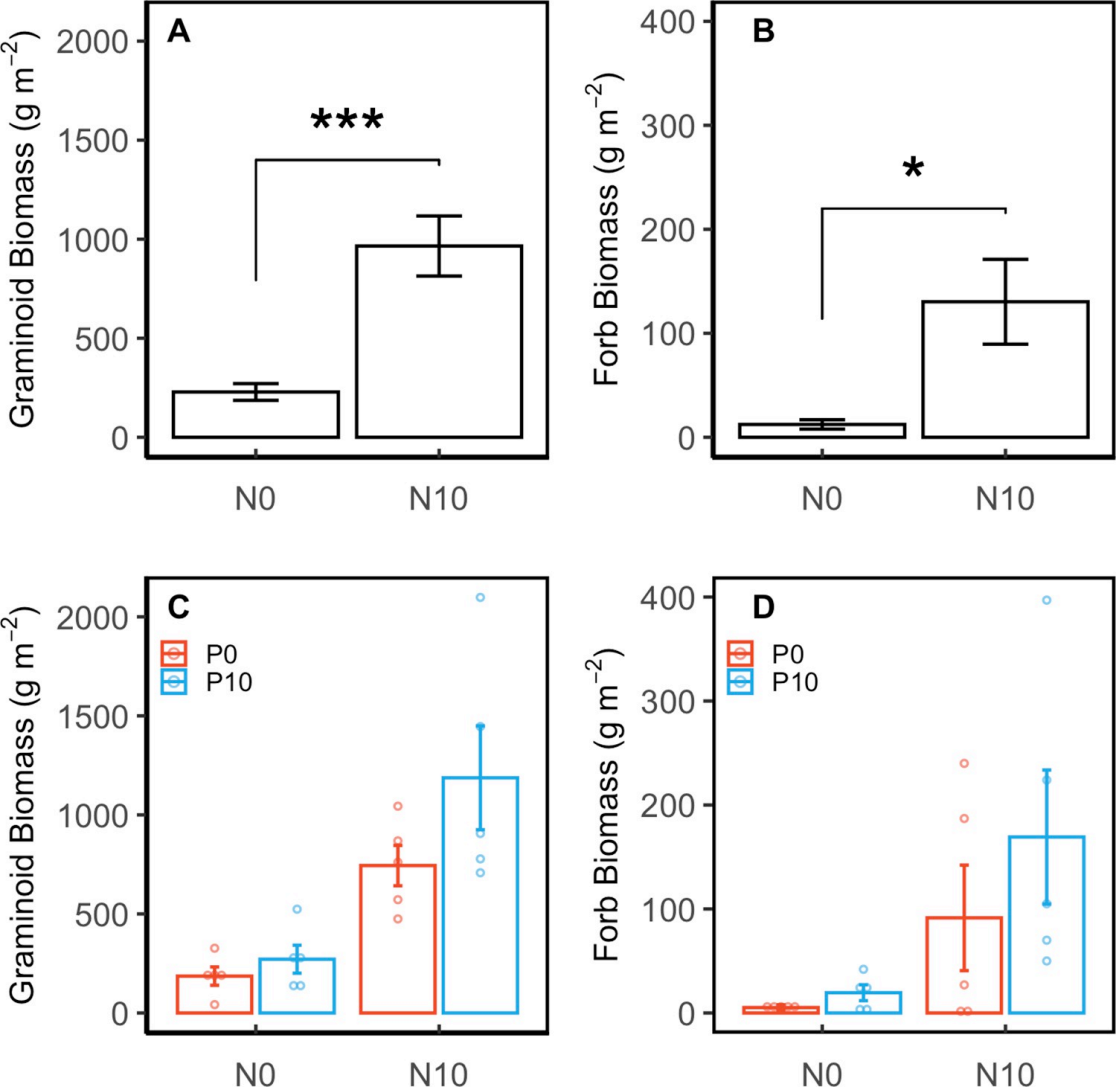
Graminoid and forb biomass production across nutrient enrichment plots. **A, B** Effect of nitrogen and phosphorus on productivity of graminoid and forb lifeforms in coastal mesic grassland (mean ± SE). Brackets and asterisks indicate significant main effect of N treatments on productivity (‘*’ *= p*-value < 0.05, ‘***’ *= p*-value < 0.0001). **C, D** Effect each nutrient treatment had on productivity of graminoids and forbs (mean ± SE). Hollow points represent productivity values of individual replicates in each treatment. Treatments include control (N0 P0), phosphorus (N0 P10), nitrogen (N10 P0), and nitrogen + phosphorus (N10 P10). Nitrogen treatments are represented on the x-axis while P treatments are represented by color. Scale of the productivity axes are adjusted to improve visualization of variation in the data and are not directly comparable across lifeform type.

There was no significant NP interaction on graminoid or forb productivity (Table 1). However, N addition was a significant main effect whereby graminoid and forb productivity were higher in plots fertilized with N compared to plots where N addition was absent (Fig 2). Graminoid productivity averaged 966 ± 152 g m^-2^ when N enrichment was present (N: 744 ± 102 and NP: 1187 ± 262 g m^-2^) and 229 ± 42 g m^-2^ (C: 186 ± 46 and P: 271 ± 71 g m^-2^) when N enrichment was absent (Fig 1). Forb productivity followed a similar pattern, when N addition was present productivity averaged 152 ± 41 g m^-2^ (N: 92 ± 51 and NP: 169 ± 64 g m^-2^) and was much lower when N fertilization was absent, averaging 12 ± 5 g m^-2^ (C: 5 ± 3 and P: 20 ± 8 g m^-2^) (Fig 1).

**Table 1:** Effects of nitrogen, phosphorus, and nitrogen + phosphorus additions on graminoid and forb productivity compared to control based on two-way ANOVA.

Treatment	Graminoid			Forb		
	MS	*F*_1, 16_	*p*-value	MS	*F*_1, 16_	*p*-value
Nitrogen (N10 P0)	11.63	37.78	**< 0.0001**	45.87	7.03	**0.0174**
Phosphorus (N0 P10)	0.91	2.95	0.1053	11.83	1.82	0.1967
Nitrogen x phosphorus (N10 P10)	0.00	0.001	0.9734	0.65	0.10	0.7561

Shown are degrees of freedom (*df*), mean square (MS), *F*-values (*F*), and *p*-values. Significant differences (*p*-value < 0.05) are bolded.

Despite differences in graminoid and forb aboveground productivity across N enriched treatments, there was no significant interaction or main effects found in belowground productivity among nutrient enrichment treatments (S1 Fig).

### Community structure, composition, and organization

There were 22 different plant species identified across all plots consisting of 10 graminoid species and 12 forb species (Table 2). No significant N x P interaction, nor N or P main effects were identified for species diversity or species richness (Table 3). Nitrogen had a significant effect on species evenness with no evidence of an N x P interactions or main effect of P (Table 3). Mean species evenness was significantly higher in N treated communities compared to communities without N treatments (ANOVA: F_1, 16_ = 9.98, *p*-value < 0.05).

**Table 2:** Species information with NMDS correlations along axis 1 and 2 and results from envfit randomization test.

Species	Lifeform	NMDS1	NMDS2	*p*-value (envfit)
*Ammophila breviligulata*	Graminoid	-0.65	0.23	0.004*
*Andropogon virginicus*	Graminoid	0.44	0.81	0.001*
*Euphorbia maculata*	Forb	0.05	0.17	0.768
*Conyza canadensis*	Forb	0.32	-0.76	0.002*
*Cyperus esculentus*	Graminoid	-0.75	-0.18	0.001*
*Panicum acuminatum*	Graminoid	0.58	0.01	0.060
*Dysphania ambrosioides*	Forb	-0.07	-0.13	0.817
*Festuca rubra*	Graminoid	0.18	-0.15	0.622
*Fimbristylis castanea*	Graminoid	0.47	0.48	0.016*
*Gnaphalium purpureum*	Forb	0.34	-0.61	0.010*
*Krigia caespitosa*	Forb	-0.04	0.14	0.843
*Lepidium virginicum*	Forb	-0.26	-0.05	0.568
*Linum virginianum*	Forb	0.50	0.47	0.022*
*Monarda punctata*	Forb	-0.34	-0.06	0.315
*Oenothera humifusa*	Forb	0.20	-0.41	0.165
*Panicum amarum*	Graminoid	-0.42	-0.52	0.007*
*Panicum dichotomiflorum*	Graminoid	-0.17	0.09	0.720
*Physalis walteri*	Forb	-0.35	0.05	0.305
*Sabatia stellaris*	Forb	0.51	0.46	0.066
*Setaria parviflora*	Graminoid	-0.56	0.49	0.001*
*Solidago sempervirens*	Forb	-0.51	0.00	0.083
*Spartina patens*	Graminoid	-0.57	0.40	0.010*

Bold represents significance (*p-*value < 0.05).

**Table 3:** Effects of nutrient additions on plant community metrics (richness, evenness, and Shannon’s diversity index [*H′*]).

Treatment	Richness	Evenness	*H′*
Control (N0 P0)	9.8 ± 0.6	0.42 ± 0.02	1.74 ± 0.06
Phosphorus (N0 P10)	10.4 ± 0.7	0.48 ± 0.01	1.89 ± 0.07
Nitrogen (N10 P0)	10.2 ± 0.4	0.40 ± 0.10	1.83 ± 0.06
Nitrogen + Phosphorus (N10 P10)	9.0 ± 0.3	0.35 ± 0.01	1.73 ± 0.05

Significant differences (*p* < 0.05) are indicated by shared letter codes based on post-hoc pairwise comparison results. Values that do not share the same letter are significantly different.

**Fig 3:**
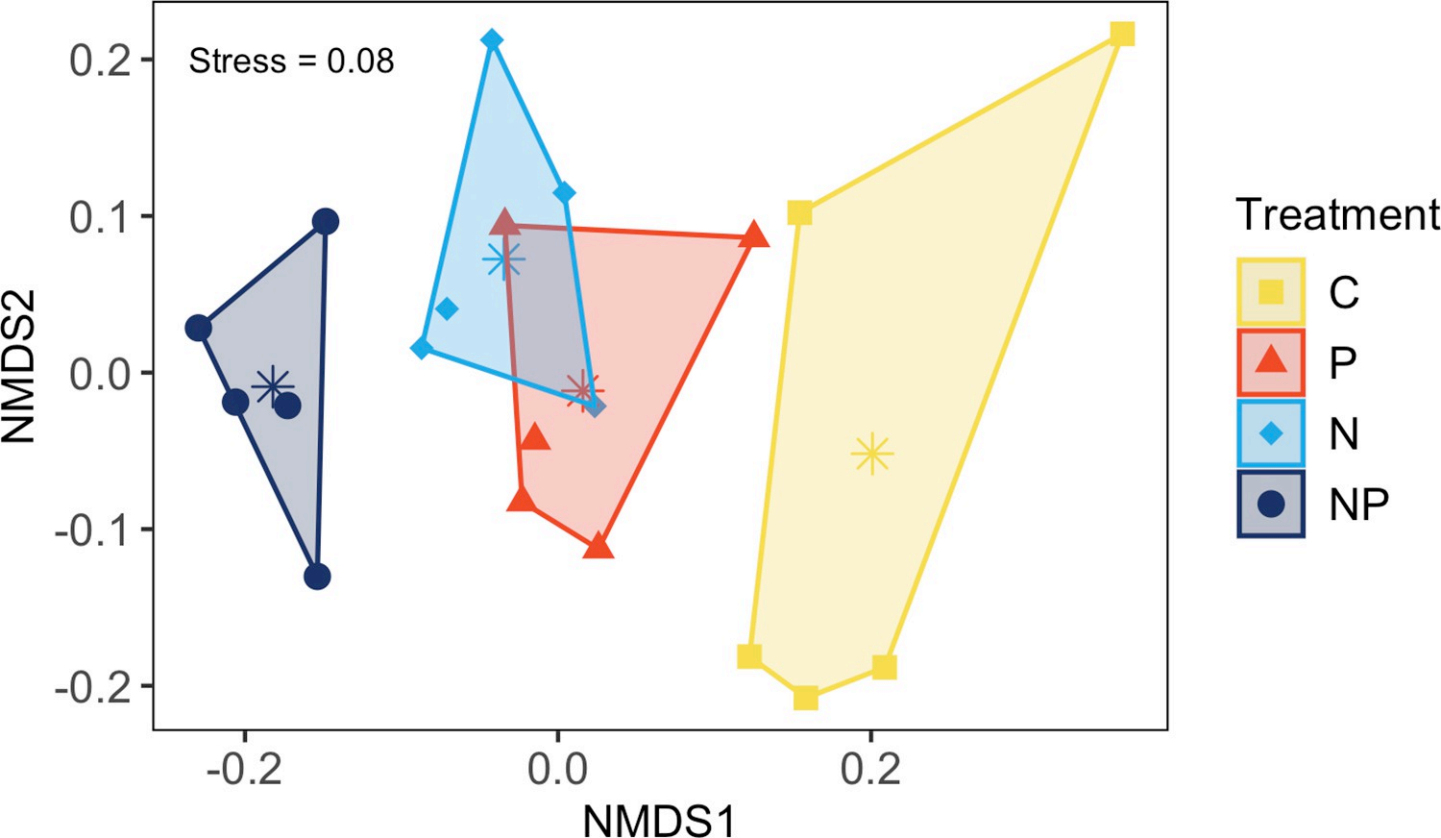
Non-metric multidimensional scaling ordination of species composition across nutrient enrichment treatments. Non-metric multidimensional scaling (NMDS) ordination of species composition grouped into convex hulls by nutrient treatment. Points represent within treatment replicates with stars indicating centroids of each treatment group. Centroids can be viewed as a representation of mean community composition. Colors and symbols are matched to nutrient treatment (C = control, P = phosphorus, N = nitrogen, NP = nitrogen + phosphorus).

The NMDS analysis on community composition of treatment groups reached a final 3-dimensional solution with a minimized stress = 0.08 (Fig 3). Nutrient enrichment caused all treatments to differ in mean community composition (PERMANOVA: F_3, 16_ = 4.52, *p*-value = 0.001) (Table 4). Community composition of all treatment groups clearly varied along NMDS1 (Fig 3). Within-group dispersion did not vary, suggesting similar beta-diversity among nutrient treated communities (ANOVA: F_3, 16_ = 2.05, *p*-value > 0.05).

**Table 4:** Pairwise comparisons of plant community composition between nutrient treatments based on PERMANOVA post-hoc test.

Pairwise comparison	F-statistic	*p-*value (FDR adjusted)
Control vs Phosphorus	3.14	**0.0240**
Control vs Nitrogen	4.32	**0.0120**
Control vs Nitrogen + Phosphorus	8.89	**0.0120**
Phosphorus vs Nitrogen	2.52	**0.0348**
Phosphorus vs Nitrogen + Phosphorus	4.35	**0.0140**
Nitrogen vs Nitrogen + Phosphorus	2.77	**0.0380**

Bolded values indicate significant differences (*p*-value < 0.05).

Species correlated with NMDS1 and/or NMDS2 were used to disentangle species specific contributions to the dissimilarity of communities (Table 1). Nutrient treated communities (N, P, and NP) correlated with perennial graminoid species (*Ammophila breviligulata*, *Spartina patens*, *Cyperus esculentus*, *Panicum amarum*, and *Setaria parviflora*), suggesting higher abundance of these species in treated communities compared to control communities (Table 1). Alternatively, control communities were correlated with forb species, including *Conyza canadensis*, *Gnaphalium purpureum*, and *Linum virginianum* as well as a perennial graminoid, *Andropogon virginicus* (Table 1).

### RACs and abundance differences

**Fig 4:**
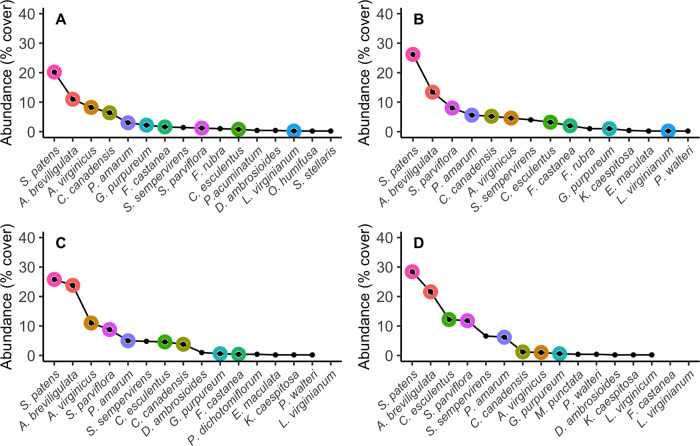
Rank abundance curves of species across nutrient enrichment treatments. Rank abundance curves (RACs) for all species in each treatment group. **A, B)** RACs for species in control and phosphorus plots, respectively. **C, D)** RACs for species in nitrogen and nitrogen + phosphorus plots, respectively. Species that were found to significantly correlate with the spread on plots in the NMDS analysis (Fig 2) are highlighted with a colored halo.

**Fig 5:**
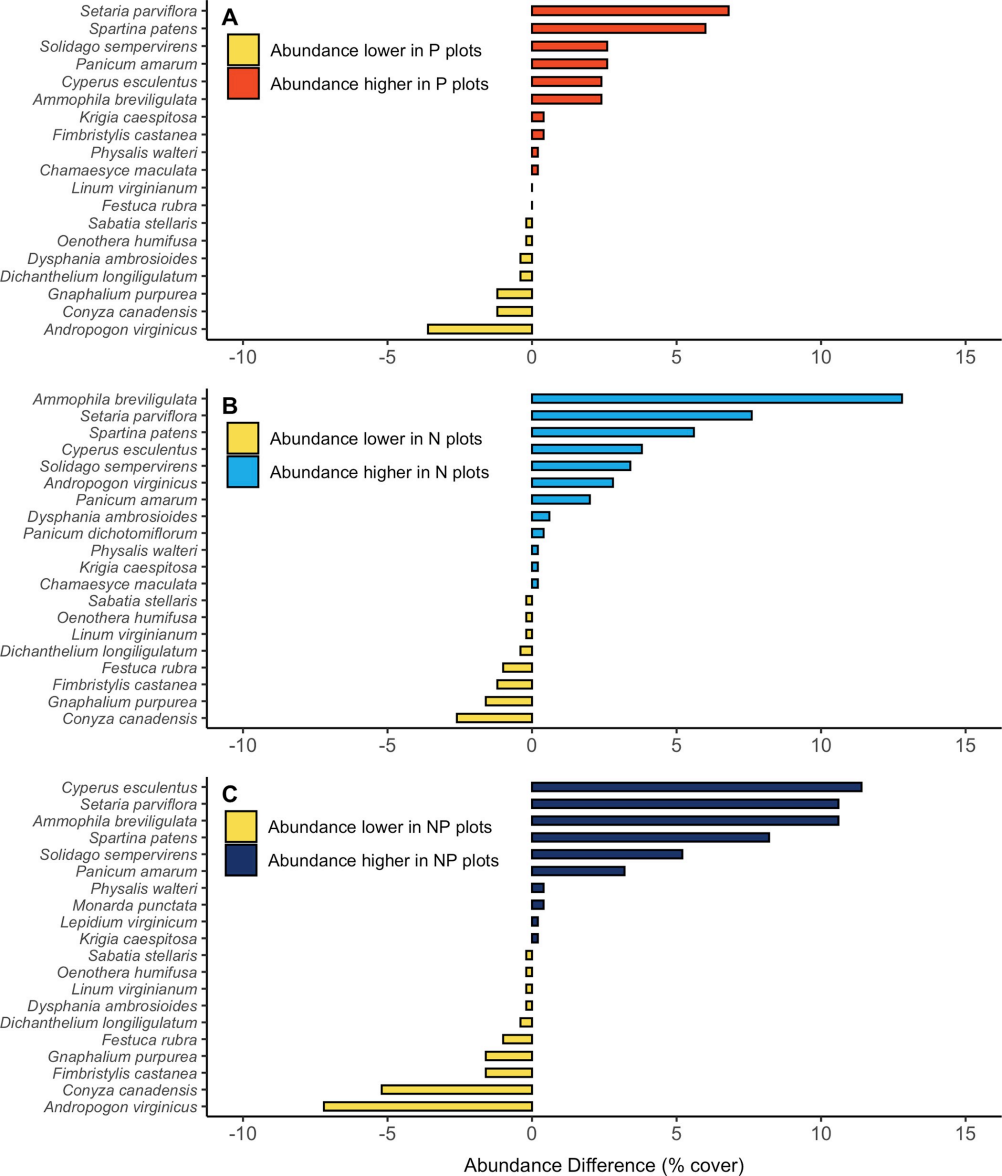
Abundance differences of species between control and each nutrient enrichment treatment. Abundance difference of each species present in **A)** control and phosphorus (P) plots; **B)** control and nitrogen (N) plots; and **C)** control and nitrogen + phosphorus (NP) plots. Control plots are used as a reference to the natural coastal grassland community. Negative values (yellow bars) indicate species that are less abundant in nutrient treated plots compared to control plots. Positive values (colors corresponding to nutrient treatment) indicate species that are more abundant in nutrient treated plots compared to control plots.

Differences in species rank order, as well as species absences, existed between treatment and control communities (Fig 4). This is especially true for species that non-randomly correlated with NMDS axes. Differences in species rank order were directly informed by abundance differences between treatment and control communities. Species similar in growth form and life history (e.g., *S*. *patens*, *A*. *breviligulata*, *S*. *parviflora*, and *C*. *esculentus*) had higher abundance across treatment communities compared to control communities (Fig 5). For some species (i.e., *S*. *patens* and *A*. *breviligulata*) higher abundance in treatment groups resulted in increased dominance, as these species did not differ in rank position across treatments (Fig 4). For example, in P treated communities *S*. *patens* abundance was on average ~6% higher and *A*. *breviligulata* was 2% higher than in control communities (Fig 5A). In N treated communities we found that *A*. *breviligulata* had abundance >10% higher compared to control communities, while *S*. *patens* was still ~6% higher (Fig 5B). In NP treated communities, we again found both *A*. *breviligulata* and *S*. *patens* had a higher abundance compared to control communities, but by larger percentages (11% and 8%, respectively) (Fig 5C).

In other cases, abundance differences were coupled with re-ordering of species ranks. For example, *S*. *parviflora* abundance was between 7–11% higher and shifted to a higher rank in all treatment communities compared to controls (Figs 4 and 5). In NP treated communities, a similar pattern was exhibited for *C*. *esculentus*, with abundance 11% higher in NP communities compared to controls, which coupled with a shift to higher species rank order (Figs 4A, 4D and 5A, 5C).

Conversely, similar species existed at lower abundance across all treatment communities compared to control communities. For example, *C*. *canadensis* and *G*. *purpureum* (annual forbs) had lower abundance in treatment communities compared to control communities (Fig 5). *Gnaphalium purpureum* abundance was consistently ~1% lower in nutrient treated communities compared to control communities. For *G*. *purpureum* this small negative difference in abundance was substantial because it caused *G*. *purpureum* to decrease between 3–5 rank positions across treatment communities compared to controls (Fig 4). *Conyza canadensis* abundance was only 1% lower in P treated communities compared to controls and resulted in a similar rank in both communities (Figs 4A, 4B and 5A). However, in N and NP treated communities, *C*. *canadensis* abundance was between 2–5% lower compared to controls which decreased the species by 4–5 rank positions (Figs 4 and 5).

Certain abundance differences, and associated species rank order, were treatment dependent. Phosphorus and NP treated communities had depressed abundance of *A*. *virginicus* compared to control communities (-4% and -7%, respectively), causing this species to decrease in rank (Figs 4 and 5). However, *A*. *virginicus* existed at the same rank in control and N treated communities and increased in abundance by ~3% in N plots (Figs 4 and 5).

Many species fluctuated in presence/absence among communities, most of which could be caused by plot spatial differences (i.e., pre-treatment distribution differences) and thus were not significantly correlated with community composition dissimilarity (Fig 4, Table 1). However, some notable species were systematically absent in N and NP treated communities. We found *L*. *virginianum* (a low-ranking species that contributes to community dissimilarity) was absent in both N and NP treated communities (Fig 4C, 4D, Table 1). In NP communities, *F*. *castanea* was absent (Fig 4D). *Fimbristylis castanea* also significantly contributed to community divergence in ordination space (Table 1).

## Discussion

### Aboveground and root productivity

Our study shows N enrichment, regardless of the P fertilization level, increases graminoid and forb productivity in our coastal grassland system. This finding was expected given the level of N limitation in coastal grassland soils [30, 33, 36]. Our results complement studies in coastal dune systems that found increased aboveground productivity following N fertilization [37, 43]. Phosphorus enrichment did not have a significant impact on graminoid productivity. These findings support those identifying N as a more limiting nutrient than P in coastal systems [45, 48–50]. We show that mid-Atlantic coastal grassland productivity is more impacted by N enrichment than P enrichment. These findings suggest that within a 3-year enrichment window, coastal grassland systems do not experience the same synergistic effects that are seen in at the global scale [1]. As a result, local scale response to nutrients should be a focal research initiative to determine how N rather than P interact with plant communities of specific habitats to influence ecosystem functioning. While our results do not show P as having significant impacts increasing productivity across vegetative lifeforms, there is a non-significant trend showing NP enrichment causing increased productivity compared to N enrichment alone. This response may reflect similarities with European coastal system response to nutrient enrichment [45], but more data is required to determine a definitive response.

Unexpectedly, we did not find difference among treatments in root productivity. We expected root productivity to increase in response to nutrient enrichment based on previous research indicating that N can have long-lasting effects on belowground biomass in coastal dune systems [43]. However, given that our study was conducted in a coastal grassland, interactions that influence plant success differ from those dominating in dune plant communities, such as increased competitive interactions and more frequent access to freshwater [30, 35]. Similar to our findings, global scale research shows that aboveground and belowground productivity patterns do not always correlate in the presence of nutrient enrichment [60]. Cleland et al. [60] suggest belowground biomass increases with nutrient enrichment (N addition), but only when coupled with high light. However, as light competition increases at the soil surface, N additions decreased belowground biomass [60]. We suggest a similar response in our grassland system. Enrichment of nutrient poor, sandy soils likely increase aboveground productivity enough to effectively decrease light at the soil surface, causing decreased belowground production. Increased aboveground biomass, resulting in competition for light and space, has also been shown to impact multiple aspects of community structure, composition, and organization which was found in our study as well.

### Community structure

Multiple aspects of plant community structure can be altered as competition for light and space increases [2, 7, 10, 11, 19]. Our results show that in a coastal grassland system, nutrient enrichment did not have impacts on species diversity or species richness. However, we did find that species evenness differed between specific nutrient enrichment treatments. Plots fertilized with N had significantly lower species evenness compared to plots that received no N treatment. This pattern is likely driven by increased dominance of the top ranked species in the community (*A*. *breviligulata* and *S*. *patens*), a well-documented response to N additions in coastal dunes [37, 61].

Nutrient enrichments can still impact community composition and organization without evident changes in common community structure metrics [4]. Here, we found nutrient enrichment treatments drive significant differences in community composition, causing distinct communities associated with each treatment. We found that when N enrichment is present productivity increases and community composition differs compared to controls, while P enrichment only influences community composition. Nutrient impacts on community composition but not productivity highlights benefits of understanding both species and functional trait response to nutrient enrichment, as both can inform different community-ecosystem relationships [8]. For studies focusing on species response, investigating abundance differences and rank order reshuffling in each community provides an outlet for detailing how increased dominance, species re-ordering, and absences of specific species ignite community differences among treatment groups.

### Community re-organization

Our data shows that N, P, and NP enrichment all result in species re-ordering of a few key species and increased dominance of top-ranking species. These findings expand on previous dune research showing N fertilization increases the dominance of certain species over time [37, 61]. *Ammophila breviligulata* and *S*. *patens* increased abundance across treatment groups but did not contribute to species re-organization, as they remained the top ranked species across treatment groups. These two graminoid species have naturally high abundance in mid-Atlantic coastal grasslands. The increase in abundance and rank order of graminoid species compared to other lifeforms has been found in many systems, as graminoids (especially those that are nitrophilous) tend to infiltrate N fertilized plots rapidly [13]. As a result, literature describes nitrophilous graminoids as “winners” in nutrient driven competitive scenarios [13]. Increased dominance is an important consideration because these species can impact ecological interactions that contribute to eliminating species that are normally able to colonize in coastal grasslands. These findings are similar to those found in dune systems [37]. Additionally, other graminoid species displayed systematic increase in our grassland site across nutrient enrichment plots. For example, *S*. *parviflora* increased abundance across all treatment groups, which caused significant re-organization of species ranks within nutrient treated communities. *Setaria parviflora* became one of the top ranked species in each nutrient enrichment treatment, shifting from 9^th^ ranked species in C plots to 3^rd^-4^th^ ranked species in nutrient enrichment plots. *Cyperus esculentus* also increased abundance across all nutrient enrichment treatments, with a major increase in NP plots, where it became the 3^rd^ most dominant species.

We also found some annual forbs and perennial graminoids systematically decrease or become entirely absent in response to nutrient enrichment, contributing to community re-organization and community composition differences. *Andropogon virginicus* decreased abundance in P and NP plots, impacting its within-community rank position which contributed to differences in community composition. Many presence/absence differences exist among low-ranking species in each treatment, however only *F*. *castanea* and *L*. *virginianum* were both significantly correlated with community divergence and also absent in N and NP plots. These results show nutrient enrichment (N, P, or NP) can cause differences among communities driven by dominant species re-ordering, while N and NP also can cause absence of key species in coastal grasslands. We posit mechanisms leading to responses found here are caused by interactions between increased productivity and characteristics of successful vs. unsuccessful species, which we highlight below.

Previous research suggests long-standing community change on barrier island dunes by altered N flux is primarily driven by competitive exclusion [37, 61]. We expand upon these findings by showing similar patterns in coastal grasslands and propose that certain characteristics of successful species promote increased abundance in nutrient enrichment plots thus altering community organization and composition. Gross and Mittelbach [62] show tall clonal species have substantial impacts on community structure in fertilized grasslands. Clonal species were found to increase in abundance after fertilization and correlated with decreased species richness driven by the ability to spread vegetatively as decreased light limited seedling recruitment [62]. In our study, species that increased dominance and rank order in fertilized plots (i.e., *S*. *patens*, *A*. *breviligulata*, *S*. *parviflora*, and *C*. *esculentus*) are clonal and do not rely on seedling recruitment in competitive environments. Furthermore, we also show lower abundance and/or rank order of *G*. *purpureum* (annual forb) and *C*. *canadensis* (annual forb) across all treatments compared to controls. As annual forbs, abundance of these species can be limited by competitive exclusion, primarily through decreased dispersal ability and seedling recruitment in densely vegetated areas.

Competitive exclusion mechanisms can also explain lower abundance and rank order of *A*. *virginicus* (short-lived, ruderal, non-rhizomatous graminoid) in NP treated communities. We suggest increased productivity in NP treated plots influences *A*. *virginicus* recruitment. Previous studies have identified decreased *A*. *virginicus* abundance as competition increases [63, 64]. Peters and Lowance [65] found *A*. *virginicus* abundance decreased in N fertilized plots, eventually leading to species replacement by other dominant graminoids. Interestingly, we found *A*. *virginicus* conserved its rank order in N treated communities, compared to controls, suggesting that abundance may not be consistently impacted across treatments. Alternatively, *A*. *virginicus* abundance may be impacted by other biotic/abiotic interactions in our system that were not measured in the current study.

Species absence was a significant factor influencing community composition differences and was most common in N and NP treated communities. While previously mentioned sub-optimal recruitment conditions can explain the absence of *L*. *virginianum* in N and NP plots, it does not explain the absence of *F*. *castanea* in NP plots. The *Fimbristylis* genera are often clonal and grow in dense caespitose tufts, which are traits that would indicate success in highly competitive environments. However, *F*. *castanea* is also an early successional species that pioneers newly developed swales and is eventually replaced by other mid- to late-successional species after 4–7 years [66]. Nutrient enrichment has been shown to increase successional rates in a number of terrestrial systems around the world including coastal dune and grassland systems [13]. Here, NP enrichment may alter rates of succession by the third year of enrichment.

## Conclusions and implications for barrier island systems

Impacts of nutrient enrichment on barrier island grassland systems has not accrued much focus despite previous work indicating community composition and productivity changes in coastal dune systems [37, 43, 61]. We show that some patterns are conserved across dune and grassland habitats such as nutrient enrichment increases aboveground graminoid biomass. However, belowground productivity has also been shown to increase in response to nutrient enrichment on barrier island dune systems [43], which we did not find in our coastal grassland site. This suggests that adjacent habitats may be influenced by nutrients in different ways. Increased aboveground growth response could decrease light levels at the soil surface in grasslands, which has been shown to stagnate N driven belowground productivity at a global scale [60].

Similar to previous dune studies, we found nutrients impact community composition and organization by increasing dominance of *S*. *patens* and *A*. *breviligulata*, enhancing our understanding of their role in grassland competitive interactions and influence over plant community organization and future trajectory. We show that previously dominant species can become more dominant after fertilization, resulting in an exclusionary effect whereby they prevent colonization and/or success of neighboring species. These types of mechanistic responses to nutrient addition disrupt coexistence within native plant communities, which elicits potential for community instability. Such effects are not normally seen in coastal grasslands because nutrient limitation serves as a stressor maintaining levels of coexistence [67]. Community composition differences without major changes in commonly measured community structure metrics (i.e., species diversity and richness) are evidence that large impacts on community composition can occur from community re-organization of species ranks without clear changes in basic community structure metrics [4, 52]. Community responses like these highlight a critical avenue for future research to use community re-organization analyses when obvious changes in species diversity, richness, or evenness are not apparent.

By investigating abundance and RAC differences in nutrient enrichment plots compared to control plots, we found increased abundance and rank order of specific species inform how nutrient enrichment alters coastal grassland community organization. Species exhibiting clonal growth strategy may be better competitors as nutrient additions increase productivity and decrease light availability, a mechanism found to decrease success of dispersal-driven annual species in other grassland systems [62]. By utilizing RAC analyses more frequently, future research can understand more nuanced mechanisms that may be impacting plant community response to nutrient enrichment.

## Supporting information

S1 Fig Rooting mass across nutrient enrichment treatments. Rooting biomass among nutrient treatments in coastal mesic grassland. Nitrogen treatment levels are labeled on the x-axis while phosphorus treatment levels are indicated by color. Treatments include control (N0P0), phosphorus (N0P10), nitrogen (N10P0), and nitrogen + phosphorus (N10P10). Dots represent individual replicates of each treatment to visualize the spread of the data.
(TIF)
