# Supplementary figures and images for: Plant community re-organization and increased productivity due to multi-year nutrient enrichment of a coastal grassland
Source: PLoS One. 2022 Jul 28;17(7):e0270798. doi: 10.1371/journal.pone.0270798 (PMC9333261; doi:10.1371/journal.pone.0270798)

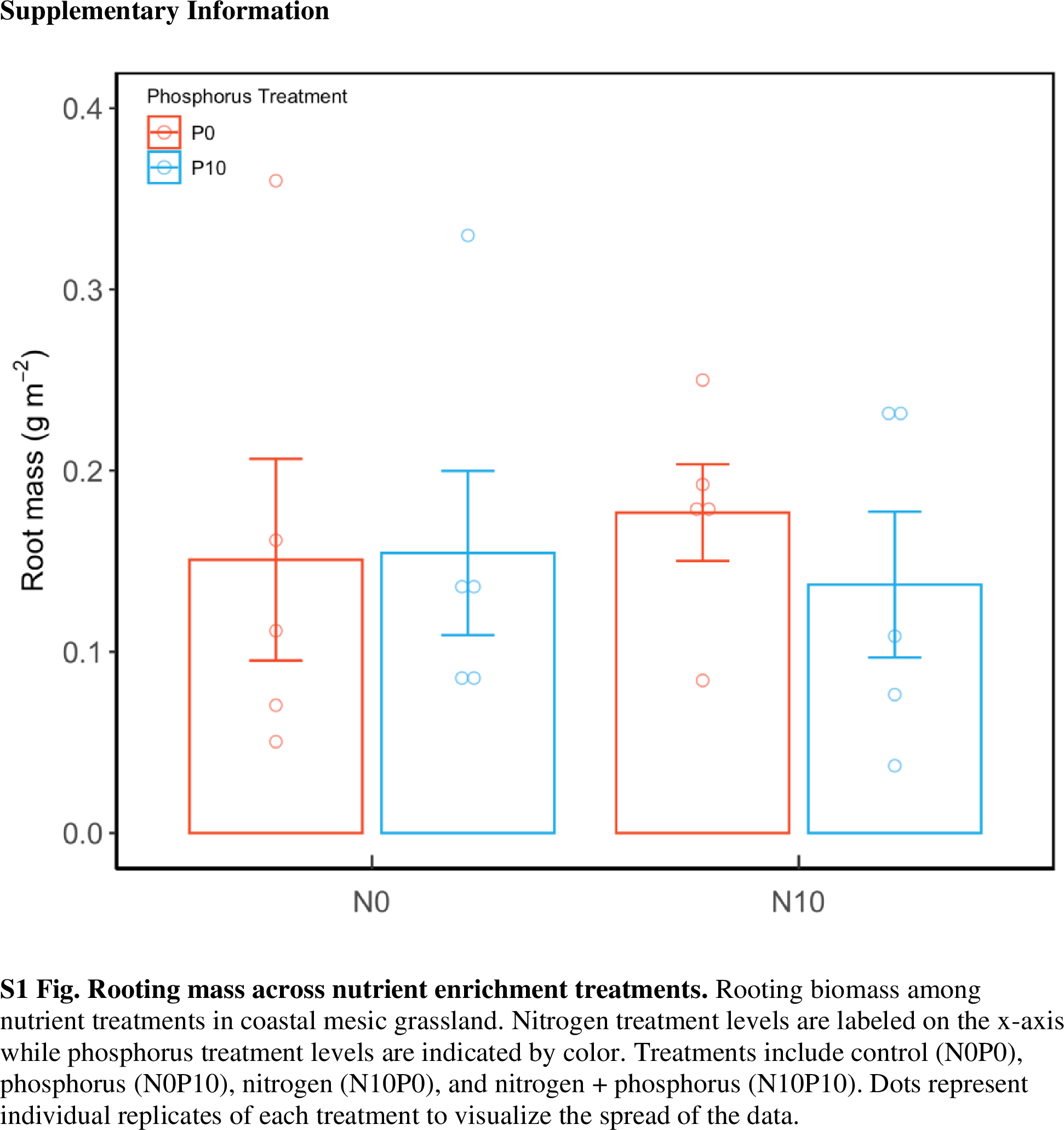

Supplement: S1 Fig — Rooting biomass among nutrient treatments in coastal mesic grassland. Nitrogen treatment levels are labeled on the x-axis while phosphorus treatment levels are indicated by color. Treatments include control (N0P0), phosphorus (N0P10), nitrogen (N10P0), and nitrogen + phosphorus (N10P10). Dots represent individual replicates of each treatment to visualize the spread of the data. (TIF) [file pone.0270798.s001.tif]
